# Ethyl 1-benzoyl-4-hy­droxy-2,6-diphenyl-1,2,5,6-tetra­hydro­pyridine-3-carboxyl­ate

**DOI:** 10.1107/S1600536811003266

**Published:** 2011-02-02

**Authors:** G. Aridoss, S. Sundaramoorthy, D. Velmurugan, Y. T. Jeong

**Affiliations:** a Department of Image Science and Engineering, Pukyong National University, Busan 608-739, Republic of Korea; b Centre of Advanced Study in Crystallography and Biophysics, University of Madras, Guindy Campus, Chennai 600 025, India

## Abstract

In the title compound, C_27_H_25_NO_4_, the tetra­hydro­pyridine ring adopts a half-chair conformation. The three phenyl rings form dihedral angles of 66.33 (7), 87.36 (8) and 36.90 (7)° with the least-squares plane through the tetra­hydro­pyridine ring. The mol­ecular conformation is stabilized by an intra­molecular O—H⋯O hydrogen bond, generating an *S*(6) motif.

## Related literature

For related structures, see: Subha Nandhini *et al.* (2003 ▶); Nithya *et al.* (2009 ▶); Aravindhan *et al.* (2009 ▶); Aridoss *et al.* (2009 ▶, 2010 ▶). For ring conformational analysis, see: Cremer & Pople (1975 ▶); Nardelli (1983 ▶).
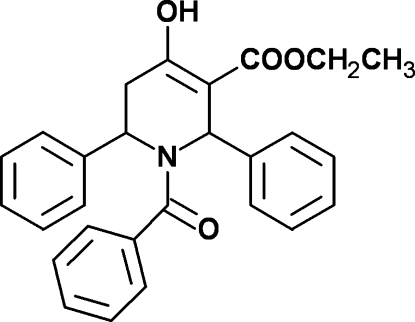


## Experimental

### 

#### Crystal data

C_27_H_25_NO_4_
*M* *_r_* = 427.48
Triclinic, 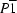

*a* = 8.2784 (7) Å
*b* = 10.6116 (9) Å
*c* = 12.7572 (11) Å
α = 85.681 (4)°
β = 89.963 (4)°
γ = 82.508 (5)°
*V* = 1107.91 (16) Å^3^
*Z* = 2
Mo *K*α radiation
μ = 0.09 mm^−1^
*T* = 293 K
0.25 × 0.23 × 0.20 mm

#### Data collection

Bruker SMART APEXII area-detector diffractometer
Absorption correction: multi-scan (*SADABS*; Bruker, 2008 ▶) *T*_min_ = 0.979, *T*_max_ = 0.983
20295 measured reflections
5526 independent reflections
4179 reflections with *I* > 2σ(*I*)
*R*_int_ = 0.024

#### Refinement

*R*[*F* ^2^ > 2σ(*F* ^2^)] = 0.043
*wR*(*F* ^2^) = 0.120
*S* = 1.04
5526 reflections
290 parameters
H-atom parameters constrained
Δρ_max_ = 0.20 e Å^−3^
Δρ_min_ = −0.15 e Å^−3^

### 

Data collection: *APEX2* (Bruker, 2008 ▶); cell refinement: *SAINT* (Bruker, 2008 ▶); data reduction: *SAINT*; program(s) used to solve structure: *SHELXS97* (Sheldrick, 2008 ▶); program(s) used to refine structure: *SHELXL97* (Sheldrick, 2008 ▶); molecular graphics: *ORTEP-3* (Farrugia, 1997 ▶); software used to prepare material for publication: *SHELXL97* and *PLATON* (Spek, 2009 ▶).

## Supplementary Material

Crystal structure: contains datablocks global, I. DOI: 10.1107/S1600536811003266/is2669sup1.cif

Structure factors: contains datablocks I. DOI: 10.1107/S1600536811003266/is2669Isup2.hkl

Additional supplementary materials:  crystallographic information; 3D view; checkCIF report

## Figures and Tables

**Table 1:** Hydrogen-bond geometry (Å, °)

*D*—H⋯*A*	*D*—H	H⋯*A*	*D*⋯*A*	*D*—H⋯*A*
O1—H1*A*⋯O2	0.82	1.85	2.570 (2)	146

**Fig. 1.**
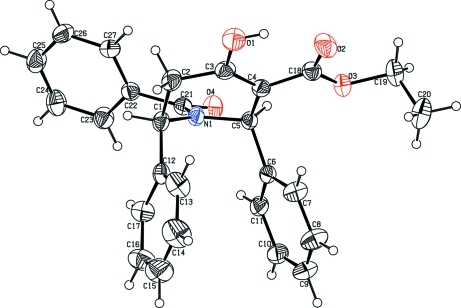
Perspective view of the title compound, showing displacement ellipsoids drawn at the 30% probability level.

**Fig. 2.**
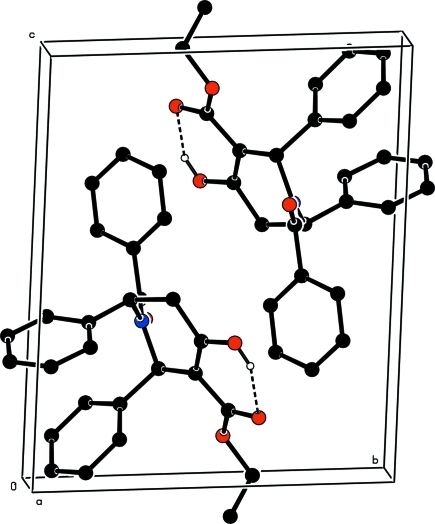
Crystal packing of the title compound, viewed down the a axis. For clarity, hydrogen atoms not involved in hydrogen bonding have been omitted.

## supplementary crystallographic information

### Comment

Owing to the relevance of piperidine-containing bioactive compounds, the development of new piperidine based derivatives continues to be a subject of considerable interest. The pharmacological effects of potential new drugs depend entirely on the stereochemistry and ring conformations of the compounds and hence the crystallographic study of the title compound has been carried out.

The *ORTEP* diagram of the title compound is shown in Fig. 1. The tetrahydropyridine ring adopts a half-chair conformation. The puckering parameters (Cremer & Pople, 1975) and the smallest displacement asymmetry parameters (Nardelli, 1983) for this ring are q_2_ = 0.344 (1) Å, q_3_ = -0.288 (1) Å; Q_T_ = 0.4485 Å and θ = 130.02 (2)°, φ_2_ = 205.6 (2)°, respectively. The three phenyl rings are twisted away from the best plane of the tetrahydropyridine ring by 66.33 (7), 87.36 (8) and 36.90 (7)°, respectively. The sum of the bond angles around the atom N1 [360.09 (10)°] of the tetrahydropyridine ring in the molecule is in accordance with *sp^2^* hybridization. The ethyl acetate group shows an extended conformation [C18—O3—C19—C20 = 90.67 (2)°]. The molecular structure is stabilized by a strong O—H···O hydrogen bond, wherein, atom O1 acts as a donor to O2, generating an *S*(6) motif.

### Experimental

To a mixture of 3-carboxyethyl-2,6-diphenylpiperidin-4-one (1 equiv.) and triethylamine (1.5 equiv.) in benzene, freshly distilled benzoyl chloride in benzene was added dropwise and stirred well at room temperature until completion. The crude mass obtained by the base work upon purification and recrystallization in distilled ethanol gave fine white crystals suitable for X-ray study.

### Refinement

The C bound H atoms positioned geometrically (C—H = 0.93–0.98 Å) and allowed to ride on their parent atoms, with 1.5*U*_eq_(C) for methyl H and 1.2*U*_eq_(C) for other H atoms.

### Crystal data

**Table:**

C_27_H_25_NO_4_	*Z* = 2
*M**_r_* = 427.48	*F*(000) = 452
Triclinic, *P*1	*D*_x_ = 1.281 Mg m^−^^3^
Hall symbol: -P 1	Mo *K*α radiation, λ = 0.71073 Å
*a* = 8.2784 (7) Å	Cell parameters from 1225 reflections
*b* = 10.6116 (9) Å	θ = 1.6–28.4°
*c* = 12.7572 (11) Å	µ = 0.09 mm^−^^1^
α = 85.681 (4)°	*T* = 293 K
β = 89.963 (4)°	Block, white
γ = 82.508 (5)°	0.25 × 0.23 × 0.20 mm
*V* = 1107.91 (16) Å^3^	

### Data collection

**Table:**

Bruker SMART APEXII area-detector diffractometer	5526 independent reflections
Radiation source: fine-focus sealed tube	4179 reflections with *I* > 2σ(*I*)
graphite	*R*_int_ = 0.024
ω and φ scans	θ_max_ = 28.4°, θ_min_ = 1.6°
Absorption correction: multi-scan (*SADABS*; Bruker, 2008)	*h* = −11→11
*T*_min_ = 0.979, *T*_max_ = 0.983	*k* = −14→14
20295 measured reflections	*l* = −17→17

### Refinement

**Table:**

Refinement on *F*^2^	Primary atom site location: structure-invariant direct methods
Least-squares matrix: full	Secondary atom site location: difference Fourier map
*R*[*F*^2^ > 2σ(*F*^2^)] = 0.043	Hydrogen site location: inferred from neighbouring sites
*wR*(*F*^2^) = 0.120	H-atom parameters constrained
*S* = 1.04	*w* = 1/[σ^2^(*F*_o_^2^) + (0.0497*P*)^2^ + 0.2116*P*] where *P* = (*F*_o_^2^ + 2*F*_c_^2^)/3
5526 reflections	(Δ/σ)_max_ < 0.001
290 parameters	Δρ_max_ = 0.20 e Å^−^^3^
0 restraints	Δρ_min_ = −0.15 e Å^−^^3^

### Special details

**Table:**

Geometry. All e.s.d.'s (except the e.s.d. in the dihedral angle between two l.s. planes) are estimated using the full covariance matrix. The cell e.s.d.'s are taken into account individually in the estimation of e.s.d.'s in distances, angles and torsion angles; correlations between e.s.d.'s in cell parameters are only used when they are defined by crystal symmetry. An approximate (isotropic) treatment of cell e.s.d.'s is used for estimating e.s.d.'s involving l.s. planes.
Refinement. Refinement of *F*^2^ against ALL reflections. The weighted *R*-factor *wR* and goodness of fit *S* are based on *F*^2^, conventional *R*-factors *R* are based on *F*, with *F* set to zero for negative *F*^2^. The threshold expression of *F*^2^ > σ(*F*^2^) is used only for calculating *R*-factors(gt) *etc*. and is not relevant to the choice of reflections for refinement. *R*-factors based on *F*^2^ are statistically about twice as large as those based on *F*, and *R*- factors based on ALL data will be even larger.

### Fractional atomic coordinates and isotropic or equivalent isotropic displacement parameters (Å^2^)

**Table:**

	*x*	*y*	*z*	*U*_iso_*/*U*_eq_	
C1	0.35152 (15)	0.26735 (12)	0.41384 (9)	0.0431 (3)	
H1	0.3276	0.2416	0.4870	0.052*	
C2	0.44264 (18)	0.38303 (14)	0.41541 (11)	0.0551 (3)	
H2A	0.4025	0.4326	0.4731	0.066*	
H2B	0.5573	0.3539	0.4285	0.066*	
C3	0.42551 (17)	0.46663 (12)	0.31645 (11)	0.0483 (3)	
C4	0.31584 (15)	0.45704 (11)	0.23982 (9)	0.0421 (3)	
C5	0.19897 (14)	0.35828 (11)	0.24886 (9)	0.0382 (2)	
H5	0.0906	0.4048	0.2328	0.046*	
C6	0.22569 (15)	0.25498 (11)	0.17071 (9)	0.0410 (3)	
C7	0.34280 (18)	0.25430 (15)	0.09352 (12)	0.0585 (4)	
H7	0.4142	0.3152	0.0911	0.070*	
C8	0.3555 (2)	0.16428 (18)	0.01975 (15)	0.0758 (5)	
H8	0.4340	0.1660	−0.0324	0.091*	
C9	0.2530 (2)	0.07296 (16)	0.02343 (14)	0.0739 (5)	
H9	0.2626	0.0117	−0.0255	0.089*	
C10	0.1359 (2)	0.07199 (15)	0.09956 (12)	0.0663 (4)	
H10	0.0657	0.0102	0.1020	0.080*	
C11	0.12177 (19)	0.16256 (13)	0.17261 (11)	0.0528 (3)	
H11	0.0416	0.1614	0.2236	0.063*	
C12	0.44595 (15)	0.15211 (13)	0.36780 (10)	0.0447 (3)	
C13	0.59156 (19)	0.15546 (17)	0.31517 (14)	0.0692 (4)	
H13	0.6367	0.2313	0.3062	0.083*	
C14	0.6700 (2)	0.0462 (2)	0.27598 (19)	0.0929 (7)	
H14	0.7676	0.0494	0.2403	0.112*	
C15	0.6072 (2)	−0.0660 (2)	0.28857 (17)	0.0873 (6)	
H15	0.6602	−0.1385	0.2606	0.105*	
C16	0.4653 (2)	−0.07137 (17)	0.34282 (16)	0.0748 (5)	
H16	0.4226	−0.1482	0.3531	0.090*	
C17	0.38547 (18)	0.03709 (14)	0.38216 (12)	0.0573 (4)	
H17	0.2892	0.0326	0.4190	0.069*	
C18	0.29783 (17)	0.55330 (12)	0.15177 (10)	0.0477 (3)	
C19	0.1465 (2)	0.63209 (17)	−0.00422 (12)	0.0685 (4)	
H19A	0.1880	0.7112	0.0078	0.082*	
H19B	0.0311	0.6514	−0.0203	0.082*	
C20	0.2327 (2)	0.5731 (2)	−0.09393 (13)	0.0832 (6)	
H20A	0.3479	0.5599	−0.0799	0.125*	
H20B	0.2108	0.6286	−0.1567	0.125*	
H20C	0.1954	0.4927	−0.1033	0.125*	
C21	0.04496 (15)	0.30444 (11)	0.40464 (10)	0.0416 (3)	
C22	0.03654 (15)	0.27946 (12)	0.52150 (10)	0.0433 (3)	
C23	−0.04315 (18)	0.18014 (14)	0.56215 (11)	0.0549 (3)	
H23	−0.0836	0.1265	0.5172	0.066*	
C24	−0.0628 (2)	0.16050 (17)	0.66984 (12)	0.0655 (4)	
H24	−0.1142	0.0925	0.6971	0.079*	
C25	−0.0065 (2)	0.24121 (17)	0.73612 (12)	0.0643 (4)	
H25	−0.0203	0.2280	0.8083	0.077*	
C26	0.0700 (2)	0.34139 (15)	0.69651 (12)	0.0620 (4)	
H26	0.1068	0.3965	0.7417	0.074*	
C27	0.09253 (18)	0.36049 (13)	0.58943 (11)	0.0531 (3)	
H27	0.1454	0.4280	0.5628	0.064*	
N1	0.19330 (12)	0.30648 (9)	0.35920 (7)	0.0388 (2)	
O1	0.52511 (14)	0.55669 (10)	0.31367 (9)	0.0663 (3)	
H1A	0.5089	0.6028	0.2592	0.099*	
O2	0.38767 (14)	0.63583 (10)	0.13602 (9)	0.0687 (3)	
O3	0.17091 (12)	0.54509 (9)	0.08975 (7)	0.0530 (2)	
O4	−0.08244 (11)	0.32515 (10)	0.35338 (8)	0.0563 (3)	

### Atomic displacement parameters (Å^2^)

**Table:**

	*U*^11^	*U*^22^	*U*^33^	*U*^12^	*U*^13^	*U*^23^
C1	0.0447 (6)	0.0496 (7)	0.0359 (6)	−0.0114 (5)	−0.0075 (5)	−0.0002 (5)
C2	0.0608 (8)	0.0598 (8)	0.0487 (7)	−0.0218 (7)	−0.0139 (6)	−0.0056 (6)
C3	0.0527 (7)	0.0446 (7)	0.0511 (7)	−0.0167 (6)	−0.0007 (6)	−0.0096 (5)
C4	0.0474 (7)	0.0384 (6)	0.0417 (6)	−0.0098 (5)	0.0013 (5)	−0.0042 (5)
C5	0.0418 (6)	0.0381 (6)	0.0350 (6)	−0.0079 (5)	−0.0044 (5)	−0.0006 (4)
C6	0.0457 (6)	0.0398 (6)	0.0372 (6)	−0.0047 (5)	−0.0091 (5)	−0.0016 (5)
C7	0.0539 (8)	0.0606 (9)	0.0643 (9)	−0.0121 (7)	0.0074 (7)	−0.0191 (7)
C8	0.0766 (11)	0.0824 (12)	0.0721 (11)	−0.0086 (9)	0.0160 (9)	−0.0333 (9)
C9	0.0989 (13)	0.0620 (10)	0.0636 (10)	−0.0070 (9)	−0.0032 (9)	−0.0282 (8)
C10	0.0967 (12)	0.0514 (8)	0.0556 (9)	−0.0254 (8)	−0.0139 (8)	−0.0085 (7)
C11	0.0701 (9)	0.0494 (7)	0.0417 (7)	−0.0193 (6)	−0.0052 (6)	−0.0026 (5)
C12	0.0398 (6)	0.0529 (7)	0.0400 (6)	−0.0036 (5)	−0.0081 (5)	0.0025 (5)
C13	0.0483 (8)	0.0712 (10)	0.0847 (12)	−0.0031 (7)	0.0098 (8)	0.0087 (9)
C14	0.0584 (10)	0.0983 (16)	0.1135 (17)	0.0163 (10)	0.0255 (10)	0.0017 (13)
C15	0.0693 (12)	0.0831 (13)	0.1018 (15)	0.0267 (10)	−0.0038 (10)	−0.0202 (11)
C16	0.0639 (10)	0.0571 (9)	0.1021 (14)	0.0026 (8)	−0.0138 (9)	−0.0147 (9)
C17	0.0485 (8)	0.0558 (8)	0.0685 (9)	−0.0080 (6)	0.0002 (7)	−0.0080 (7)
C18	0.0531 (7)	0.0427 (7)	0.0476 (7)	−0.0077 (6)	0.0080 (6)	−0.0022 (5)
C19	0.0756 (10)	0.0728 (10)	0.0521 (8)	−0.0055 (8)	−0.0031 (7)	0.0214 (7)
C20	0.0807 (12)	0.1220 (16)	0.0467 (9)	−0.0197 (11)	0.0016 (8)	0.0062 (9)
C21	0.0462 (7)	0.0372 (6)	0.0424 (6)	−0.0089 (5)	−0.0010 (5)	−0.0027 (5)
C22	0.0443 (6)	0.0419 (6)	0.0430 (6)	−0.0025 (5)	0.0029 (5)	−0.0030 (5)
C23	0.0579 (8)	0.0568 (8)	0.0518 (8)	−0.0168 (6)	0.0020 (6)	−0.0002 (6)
C24	0.0652 (9)	0.0736 (10)	0.0566 (9)	−0.0149 (8)	0.0087 (7)	0.0130 (8)
C25	0.0656 (9)	0.0780 (11)	0.0440 (8)	0.0082 (8)	0.0107 (7)	−0.0014 (7)
C26	0.0753 (10)	0.0596 (9)	0.0489 (8)	0.0073 (7)	−0.0004 (7)	−0.0188 (7)
C27	0.0654 (9)	0.0428 (7)	0.0514 (8)	−0.0055 (6)	0.0025 (6)	−0.0085 (6)
N1	0.0415 (5)	0.0405 (5)	0.0347 (5)	−0.0073 (4)	−0.0034 (4)	−0.0013 (4)
O1	0.0744 (7)	0.0620 (6)	0.0700 (7)	−0.0369 (5)	−0.0074 (5)	−0.0056 (5)
O2	0.0769 (7)	0.0583 (6)	0.0731 (7)	−0.0273 (5)	0.0042 (6)	0.0116 (5)
O3	0.0599 (6)	0.0546 (5)	0.0427 (5)	−0.0073 (4)	−0.0006 (4)	0.0081 (4)
O4	0.0445 (5)	0.0718 (7)	0.0530 (6)	−0.0128 (5)	−0.0049 (4)	0.0030 (5)

### Geometric parameters (Å, °)

**Table:**

C1—N1	1.4798 (15)	C14—H14	0.9300
C1—C12	1.5197 (19)	C15—C16	1.369 (3)
C1—C2	1.5237 (18)	C15—H15	0.9300
C1—H1	0.9800	C16—C17	1.379 (2)
C2—C3	1.4831 (19)	C16—H16	0.9300
C2—H2A	0.9700	C17—H17	0.9300
C2—H2B	0.9700	C18—O2	1.2263 (16)
C3—O1	1.3397 (15)	C18—O3	1.3315 (17)
C3—C4	1.3531 (18)	C19—O3	1.4545 (16)
C4—C18	1.4543 (18)	C19—C20	1.484 (2)
C4—C5	1.5145 (16)	C19—H19A	0.9700
C5—N1	1.4751 (14)	C19—H19B	0.9700
C5—C6	1.5315 (16)	C20—H20A	0.9600
C5—H5	0.9800	C20—H20B	0.9600
C6—C7	1.3812 (19)	C20—H20C	0.9600
C6—C11	1.3852 (18)	C21—O4	1.2276 (15)
C7—C8	1.385 (2)	C21—N1	1.3597 (16)
C7—H7	0.9300	C21—C22	1.4976 (17)
C8—C9	1.367 (3)	C22—C23	1.3833 (19)
C8—H8	0.9300	C22—C27	1.3863 (18)
C9—C10	1.372 (3)	C23—C24	1.387 (2)
C9—H9	0.9300	C23—H23	0.9300
C10—C11	1.383 (2)	C24—C25	1.371 (2)
C10—H10	0.9300	C24—H24	0.9300
C11—H11	0.9300	C25—C26	1.372 (2)
C12—C17	1.3782 (19)	C25—H25	0.9300
C12—C13	1.383 (2)	C26—C27	1.382 (2)
C13—C14	1.381 (3)	C26—H26	0.9300
C13—H13	0.9300	C27—H27	0.9300
C14—C15	1.359 (3)	O1—H1A	0.8200
			
N1—C1—C12	111.53 (9)	C14—C15—C16	119.40 (17)
N1—C1—C2	108.76 (10)	C14—C15—H15	120.3
C12—C1—C2	114.90 (11)	C16—C15—H15	120.3
N1—C1—H1	107.1	C15—C16—C17	120.01 (18)
C12—C1—H1	107.1	C15—C16—H16	120.0
C2—C1—H1	107.1	C17—C16—H16	120.0
C3—C2—C1	113.62 (10)	C12—C17—C16	121.14 (15)
C3—C2—H2A	108.8	C12—C17—H17	119.4
C1—C2—H2A	108.8	C16—C17—H17	119.4
C3—C2—H2B	108.8	O2—C18—O3	122.55 (12)
C1—C2—H2B	108.8	O2—C18—C4	124.16 (13)
H2A—C2—H2B	107.7	O3—C18—C4	113.28 (11)
O1—C3—C4	123.70 (12)	O3—C19—C20	109.75 (14)
O1—C3—C2	112.47 (11)	O3—C19—H19A	109.7
C4—C3—C2	123.74 (11)	C20—C19—H19A	109.7
C3—C4—C18	118.69 (11)	O3—C19—H19B	109.7
C3—C4—C5	122.01 (11)	C20—C19—H19B	109.7
C18—C4—C5	118.95 (11)	H19A—C19—H19B	108.2
N1—C5—C4	109.42 (9)	C19—C20—H20A	109.5
N1—C5—C6	113.34 (9)	C19—C20—H20B	109.5
C4—C5—C6	115.47 (10)	H20A—C20—H20B	109.5
N1—C5—H5	105.9	C19—C20—H20C	109.5
C4—C5—H5	105.9	H20A—C20—H20C	109.5
C6—C5—H5	105.9	H20B—C20—H20C	109.5
C7—C6—C11	118.09 (12)	O4—C21—N1	122.22 (11)
C7—C6—C5	122.82 (11)	O4—C21—C22	118.89 (11)
C11—C6—C5	118.96 (11)	N1—C21—C22	118.87 (11)
C6—C7—C8	121.02 (14)	C23—C22—C27	119.20 (13)
C6—C7—H7	119.5	C23—C22—C21	118.91 (12)
C8—C7—H7	119.5	C27—C22—C21	121.59 (12)
C9—C8—C7	120.12 (16)	C22—C23—C24	120.09 (14)
C9—C8—H8	119.9	C22—C23—H23	120.0
C7—C8—H8	119.9	C24—C23—H23	120.0
C8—C9—C10	119.73 (14)	C25—C24—C23	120.07 (15)
C8—C9—H9	120.1	C25—C24—H24	120.0
C10—C9—H9	120.1	C23—C24—H24	120.0
C9—C10—C11	120.26 (15)	C24—C25—C26	120.31 (14)
C9—C10—H10	119.9	C24—C25—H25	119.8
C11—C10—H10	119.9	C26—C25—H25	119.8
C10—C11—C6	120.77 (14)	C25—C26—C27	120.03 (14)
C10—C11—H11	119.6	C25—C26—H26	120.0
C6—C11—H11	119.6	C27—C26—H26	120.0
C17—C12—C13	118.23 (14)	C26—C27—C22	120.27 (14)
C17—C12—C1	118.10 (12)	C26—C27—H27	119.9
C13—C12—C1	123.64 (13)	C22—C27—H27	119.9
C14—C13—C12	120.02 (17)	C21—N1—C5	118.15 (10)
C14—C13—H13	120.0	C21—N1—C1	124.93 (10)
C12—C13—H13	120.0	C5—N1—C1	116.82 (9)
C15—C14—C13	121.17 (18)	C3—O1—H1A	109.5
C15—C14—H14	119.4	C18—O3—C19	118.01 (12)
C13—C14—H14	119.4		
			
N1—C1—C2—C3	−39.33 (16)	C1—C12—C17—C16	−179.77 (14)
C12—C1—C2—C3	86.47 (15)	C15—C16—C17—C12	0.1 (3)
C1—C2—C3—O1	−170.79 (12)	C3—C4—C18—O2	8.2 (2)
C1—C2—C3—C4	12.3 (2)	C5—C4—C18—O2	−178.52 (12)
O1—C3—C4—C18	−3.4 (2)	C3—C4—C18—O3	−170.63 (12)
C2—C3—C4—C18	173.15 (13)	C5—C4—C18—O3	2.68 (17)
O1—C3—C4—C5	−176.51 (12)	O4—C21—C22—C23	57.04 (17)
C2—C3—C4—C5	0.1 (2)	N1—C21—C22—C23	−124.55 (13)
C3—C4—C5—N1	15.95 (16)	O4—C21—C22—C27	−116.66 (15)
C18—C4—C5—N1	−157.13 (11)	N1—C21—C22—C27	61.75 (17)
C3—C4—C5—C6	−113.35 (13)	C27—C22—C23—C24	−1.6 (2)
C18—C4—C5—C6	73.57 (14)	C21—C22—C23—C24	−175.44 (13)
N1—C5—C6—C7	−130.38 (13)	C22—C23—C24—C25	1.5 (2)
C4—C5—C6—C7	−3.03 (17)	C23—C24—C25—C26	−0.3 (2)
N1—C5—C6—C11	53.88 (14)	C24—C25—C26—C27	−0.8 (2)
C4—C5—C6—C11	−178.77 (11)	C25—C26—C27—C22	0.6 (2)
C11—C6—C7—C8	0.4 (2)	C23—C22—C27—C26	0.5 (2)
C5—C6—C7—C8	−175.42 (14)	C21—C22—C27—C26	174.22 (13)
C6—C7—C8—C9	−1.0 (3)	O4—C21—N1—C5	11.51 (17)
C7—C8—C9—C10	1.0 (3)	C22—C21—N1—C5	−166.85 (10)
C8—C9—C10—C11	−0.3 (3)	O4—C21—N1—C1	−172.21 (11)
C9—C10—C11—C6	−0.4 (2)	C22—C21—N1—C1	9.43 (17)
C7—C6—C11—C10	0.3 (2)	C4—C5—N1—C21	129.05 (11)
C5—C6—C11—C10	176.29 (13)	C6—C5—N1—C21	−100.50 (12)
N1—C1—C12—C17	−68.07 (15)	C4—C5—N1—C1	−47.53 (13)
C2—C1—C12—C17	167.57 (11)	C6—C5—N1—C1	82.92 (12)
N1—C1—C12—C13	113.98 (14)	C12—C1—N1—C21	116.28 (12)
C2—C1—C12—C13	−10.38 (18)	C2—C1—N1—C21	−115.98 (13)
C17—C12—C13—C14	1.8 (2)	C12—C1—N1—C5	−67.39 (13)
C1—C12—C13—C14	179.77 (16)	C2—C1—N1—C5	60.35 (13)
C12—C13—C14—C15	−0.4 (3)	O2—C18—O3—C19	4.6 (2)
C13—C14—C15—C16	−1.3 (3)	C4—C18—O3—C19	−176.56 (12)
C14—C15—C16—C17	1.4 (3)	C20—C19—O3—C18	90.67 (17)
C13—C12—C17—C16	−1.7 (2)		

### Hydrogen-bond geometry (Å, °)

**Table:**

*D*—H···*A*	*D*—H	H···*A*	*D*···*A*	*D*—H···*A*
O1—H1A···O2	0.82	1.85	2.570 (2)	146
